# N-Acetyl-Cysteine as Effective and Safe Chelating Agent in Metal-on-Metal Hip-Implanted Patients: Two Cases

**DOI:** 10.1155/2016/8682737

**Published:** 2016-04-11

**Authors:** Andrea Giampreti, Davide Lonati, Benedetta Ragghianti, Anna Ronchi, Valeria Margherita Petrolini, Sarah Vecchio, Carlo Alessandro Locatelli

**Affiliations:** ^1^Pavia Poison Centre and National Toxicology Information Centre, IRCCS Maugeri Foundation Clinical Institute and University of Pavia, 27100 Pavia, Italy; ^2^Diabetes Agency, Careggi Teaching Hospital, 50134 Firenze, Italy

## Abstract

Systemic toxicity associated with cobalt (Co) and chromium (Cr) containing metal hip alloy may result in neuropathy, cardiomyopathy, and hypothyroidism. However clinical management concerning chelating therapy is still debated in literature. Here are described two metal-on-metal hip-implanted patients in which N-acetyl-cysteine decreased elevated blood metal levels. A 67-year-old male who underwent Co/Cr hip implant in September 2009 referred to our Poison Control Centre for persisting elevated Co/Cr blood levels (from March 2012 to November 2014). After receiving oral high-dose N-acetyl-cysteine, Co/Cr blood concentrations dropped by 86% and 87% of the prechelation levels, respectively, and persisted at these latter concentrations during the following 6 months of follow-up. An 81-year-old female who underwent Co/Cr hip implant in January 2007 referred to our Centre for detection of high Co and Cr blood levels in June 2012. No hip revision was indicated. After a therapy with oral high-dose N-acetyl-cysteine Co/Cr blood concentrations decreased of 45% and 24% of the prechelation levels. Chelating agents reported in hip-implanted patients (EDTA, DMPS, and BAL) are described in few cases. N-acetyl-cysteine may provide chelating sites for metals and in our cases reduced Co and Cr blood levels and resulted well tolerable.

## 1. Introduction

Safety concerns regarding wear and corrosion of cobalt/chromium (Co/Cr) containing hip prosthetics and subsequent Co/Cr release have resulted in products recall, public alert, and need for clinicians, health authorities, and scientific societies to evaluate and follow up metal-on-metal (MOM) hip-implanted patients for local and systemic toxic effects [1]. However only few reports of systemic toxicity from metals released from hip implant have been described in the literature [2]. Moreover consensus statement and evidence based data on management for MOM hip arthroplasty are mainly focused on hip implant-related problems as implant failure, local metallosis, and hip pain; nowadays minor attention is focused on toxicological management [3–5]. Regulatory agencies that tried to rule the management and follow-up of patients with metal hip implant focused mainly on orthopedic evaluation, imaging monitoring (e.g., Magnetic Resonance Imaging in Metal Artifact Reduction (MARS) techniques, CT scan, and hip echography), and metal blood tests. Toxicologic evaluation and decision concerning possible specific therapy, when chelating therapy should be evaluated, or which chelating agent should be chosen are often less treated and more debated topics [6]. In particular no data and no clinical experience exist concerning chelating therapy in asymptomatic MOM hip-implanted patients in which blood cobalt levels are elevated. We describe two MOM hip-implanted patients in which mildly elevated blood cobalt levels were associated with absence of systemic manifestations of cobaltism and chelating therapy with N-acetyl-cysteine (NAC) resulted effective and safe in decreasing Co/Cr blood levels.

## 2. Case Reports

**Figure 1:**
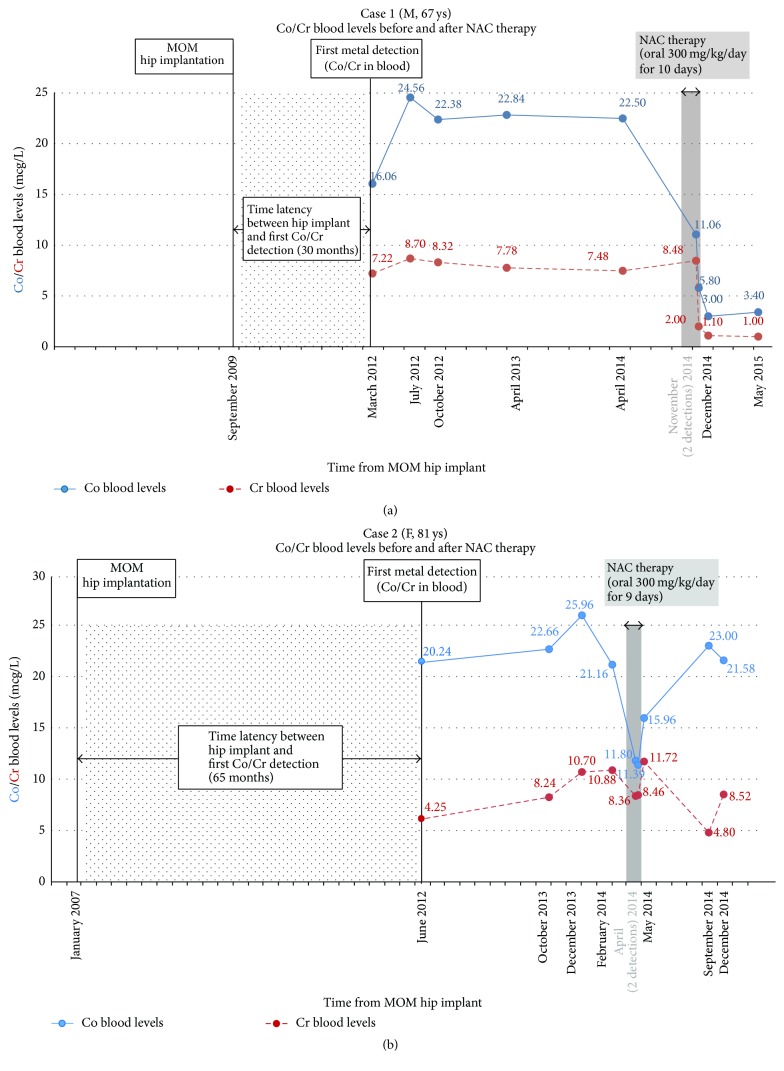
(a) Cobalt (Co) and chromium (Cr) blood levels in case  1. In the figure are reported time latency from MOM implant and first metal detection and metal blood concentrations before, during, and after chelation treatment with high-dose oral NAC. (b) Cobalt (Co) and chromium (Cr) blood levels in case  2. In the figure are reported time latency from MOM implant and first metal detection and metal blood concentrations before, during, and after chelation treatment with high-dose oral NAC.

*Case  1*. In May 2012, a 67-year-old male patient was referred to our Poison Control Centre for detection of high Co (16.06 mcg/L; normal value < 0.9) and Cr (7.22 mcg/L; normal value < 0.5) blood levels in March 2012 (Figure 1(a)). His past medical history was positive only for previous cochlear implant in 2005 and hypertension in treatment with candesartan cilexetil and hydrochlorothiazide. In September 2009, the patient underwent MOM Co/Cr alloy total hip implantation for left coxarthrosis (DePuy ASR*™* XL Hip System). The postoperative clinical course was good with normal range of movement and no local pain. Except for Co/Cr blood levels, laboratory exams (blood chemistry and thyroid function) and instrumental evaluation (echocardiography and electromyography/electroneurography at superior and inferior limbs) resulted normal. An orthopedic evaluation evidenced no signs or symptoms of implant failure and an echography performed on March 2012 resulted negative for local reactions or massive fluid collection near the implant. Magnetic Resonance Imaging in MARS techniques performed in November 2012 evidenced a little fluid collection near the acetabular cup that progressively reduced at the control in March 2013 and April and December 2014. Considering the presence of elevated Co/Cr blood levels in absence of other local and systemic manifestations the patient underwent metal monitoring on blood samples for the following three years (Figure 1(a)). Due to the persisting elevated Co/Cr blood levels (Co above 20 mcg/L and Cr above 7 mcg/L) a chelation therapy with oral high-dose NAC (300 mg/kg/day for 10 days) was performed in November 2014. NAC was well tolerated and no adverse reactions were reported. Co/Cr blood concentrations performed in December 2014 immediately after the chelation dropped by about 86% and 87% of the prechelation levels, respectively, and persisted at these latter concentrations during the following 6 months (Figure 1(a)). Also urine Co/Cr concentrations modified during NAC administration and increased by about 4- and 3-fold, respectively (Co urine levels increased from 6 to 23 mcg/L; Cr urine levels increased from 2 to 6 mcg/L). At a 3-year follow-up, the patient remained asymptomatic, with no signs of local or systemic effects and persisting low Co/Cr blood levels.

*Case  2*. In November 2013, an 81-year-old female patient was referred to our Poison Control Centre for detection of high Co (20.24 mcg/L) and Cr (4.25 mcg/L) blood levels in June 2012 (Figure 1(b)). Her past medical history was positive for COPD, systemic lupus erythematosus, moderate mitral and aortic valve regurgitation, and mild renal impairment (CKD-epi calculated creatinine clearance 52 mL/min). Euthyroid multinodular goiter, hypertension, and bilateral cataracts were also reported before implant date. In January 2007 the patient underwent MOM total left hip arthroplasty (DePuy ASR*™* Hip System) for traumatic fracture. Clinical course from 2008 to 2013 was characterized by local hip pain due to postoperative myositis ossificans associated with periprosthetic fluid collection evidenced at CT imaging and hip scintigraphy. No systemic signs or symptoms of cobaltism such as peripheral polyneuropathy, hypothyroidism, or pericardial exudate were evidenced and considering particular risk factors (extreme age and patient's comorbidities) no hip revision was indicated. Due to the persisting elevated Co/Cr blood levels (Co above 20 mcg/L and Cr above 8 mcg/L) a chelation therapy with oral high-dose NAC (300 mg/kg/day for 9 days) was performed in April 2014 (Figure 1(b)). Co/Cr blood concentrations performed during chelation therapy revealed a decrease of Co/Cr blood concentrations of 45% and 24% of the prechelation levels, respectively, and an increase in Co/Cr urine excretion (Co from 21.9 to 39.5 mcg/L; Cr from 6 to 15.6 mcg/L). No adverse reactions have been evidenced during therapy except for a slight increase in body weight and blood pressure promptly reversed with diuretic treatment (oral furosemide 20 mg). At a recent follow-up, the patient is stable, with no signs of local or systemic Co/Cr effects. The metal monitoring revealed a progressive increase of Co and Cr blood concentrations during the following 8 months after the chelation therapy (Figure 1(b)).

## 3. Discussion

In recent years, safety concerns regarding MOM hip prosthesis have been raised [1]. Local and systemic toxicity associated with Co/Cr containing metal hip alloy have been reported and mainly related to Co. Arthroprosthetic cobaltism may result in neuropathy, cardiomyopathy, and hypothyroidism with late onset from prosthesis implantation. However toxicological evaluation and clinical management concerning hip surgical revision and particularly chelating therapy are still debated [12]. The role of different chelating agents, timing of administration, and chelating efficacy seem to represent debated aspects in Co/Cr chelation and in clinical management of MOM hip-implanted patients [2, 13].

Concerning the type of prostheses it has been considered that head size and implant design may play an important role in the risk of implant wear and metal ions release. For total hip implant, small heads implant presents a little additional risk of adverse reaction to metal debris and bearings surface wear. On the other hand large head implant (36 mm head size and larger) may present a higher risk of taper wear, rim loading, and reduced bearing lubrication (particularly if associated with high abduction angle) with consequent increased risk for local adverse reaction to metal debris and systemic release of metal ions and particles [4].

Concerning systemic manifestations nowadays only few reports concerning arthroprosthetic cobaltism have been published in medical literature. Despite the fact that a clear and shared toxic threshold for systemic cobaltism is not reported, clinical manifestations as cardiotoxicity, peripheral neuropathy, and thyroid toxicity have been associated with high blood cobalt levels above 100, 250, and 500 mcg/L, respectively; on the other hand sensorineural hearing loss and ocular toxicity have been anecdotally associated with lower Co blood levels above 20 and 40 mcg/L, respectively [2, 14, 15]. In our cases, despite cobalt blood levels being above 20 mcg/L in both patients, no signs of ocular disease or sensorineural hearing worsening have been evidenced from hip implantation and a clinical management based on metal monitoring and seriated hear and visual control has been decided. Cochlear implant in case  1 and goiter and bilateral cataracts in case  2 were presented before hip implantation and no signs of clinical worsening have been detected after hip implant.

Concerning chelation therapy, Co and Cr chelation in MOM hip-implanted patients has been described in few cases. Chelating agents such as edetate calcium disodium (EDTA), sodium 2,3-dimercaptopropane sulfonate (DMPS), and dimercaprol (BAL) have been reported (Table 1) [7–10].

EDTA is used to bind metal ions in the practice of chelation therapy mainly for mercury and lead poisoning. It binds to a metal cation through its two amines and four carboxylates sites, enhancing metal elimination through renal excretion. Dimercaprol and DMPS are chelating agents principally involved in the treatment of poisoning by arsenic and polonium-210, respectively. Both are characterized by the presence in the molecular structure of two thiol groups that bind metal ions consequently excreted in the urine. Animal data have suggested that chelating sites of EDTA and thiol groups of dimercaprol and DMPS could be effective in chelating cobalt [16]. However anecdotal use in MOM hip-implanted patient has been described for EDTA (1 case), dimercaprol (1 case), and DMPS (2 cases). At present their efficacy and place in therapy for arthroprosthetic cobaltism remain anecdotal. It has also been kept in mind that potential renal, hepatic, or gastrointestinal adverse effects may result from EDTA, DMPS, and BAL administration, respectively [17–19]. Moreover all hip-implanted patients in which these agents have been administered removed the metal source of exposure; in fact they underwent hip implant revision because they presented severe clinical manifestations of systemic cobaltism and significantly elevated cobalt blood levels (Table 1).

Our cases presented Co/Cr blood levels lower than reported for patients that underwent hip revision and chelation therapy. Moreover, except for mild fluid collection near the prosthesis, no local or systemic manifestations of cobaltism have been registered. In current medical literature different published consensus statement on metal-on-metal total hip replacement and hip resurfacing establish that in asymptomatic patients with well-functioning hip implant but elevated Co levels the individual risk-benefit-ratio should be carefully considered for hip revision and no indications concerning which, how, and when a chelating approach should be evaluated are presented [3, 4]. In our cases slightly elevated blood cobalt levels, optimal clinical condition in case  1, and patient's comorbidities in case  2 seemed to be reasonable conditions to wait for hip implant revision. Due to the persisting increased Co/Cr blood levels a chelating approach has been evaluated. Clinical experience is very limited and chelation in these patients needs to be carefully evaluated. Thiol groups in N-acetyl-cysteine (NAC) may provide chelating sites for metals. Animal studies on comparative effects of repeated administration of different chelators on the distribution and excretion of Co evidenced that NAC both increases urine elimination and decreases tissues concentration of Co [16, 20]. Moreover also recent in vitro data evidence that NAC may chelate and form conjugates with cobalt [21]. NAC has been reported not only as effective and safe Co chelating agent in some animal models but also as potential Co chelator in one previously recently reported human case of severe arthroprosthetic cobaltism [11, 16, 20]. In our cases, NAC was preferred to other agents due to its potential safer profile and possible oral administration. High-dose NAC was orally administered and reduced Co and Cr blood levels under or near MHRA Co attention level of 7 mcg/L [6]. Moreover in both case  1 and case  2 an increase in urine Co and Cr levels was observed during NAC administration. In case  1 metal levels persisted to be low up to 6 months after NAC administration while in case  2 Co/Cr levels presented an increase during the following 8 months from NAC. This may be explained by a mild renal impairment associated with a longer exposure and subsequent larger accumulation of cobalt in case  2 (about 65 months) than in case  1 (about 30 months) that may induce a greater metal redistribution from deep tissues into blood. Moreover recent toxicokinetic studies hypothesized that women (as case  2) generally absorb more and excrete less Co than men (as case  1) [22, 23].

Clinical management of patients with MOM hip prosthesis is complex and is currently characterized by several debated aspects concerning surgical and toxicological management and follow-up. In patients with MOM hip implant and persisting increased Co/Cr blood levels a continuous exposure to Co and Cr cannot be excluded and a chelating approach may be considered. Also, if only in two cases, in our experience oral high-dose NAC resulted well tolerable and reduced elevated Co/Cr blood levels of MOM hip-implanted asymptomatic patients.

## Figures and Tables

**Table 1:** Chelation therapy in metal hip-implanted patients: in table patient's medical history, clinical manifestations, chelating agents, and metals blood levels before and after chelation treatment concerning the presented cases and those published in literature are reported.

Age/sex	Comorbidity	Hip type	Latency from implant to symptoms or blood metals	Clinical manifestations		Implant revision	Co/Cr blood levels acme before chelation	Chelating therapy (cycles)	Co/Cr blood levels after chelation	Reference
				Local	Systemic					
58/F	Type 2 D Hypert.	MOP^*∗*^	6–9 months	Prosthesis wear and local metallosis	Visual/hearing loss II–VII cranial nerve disorders Sensorimotor disorders Mild hypothyroidism	Yes	Co 549 mcg/L Cr 54 mcg/L	EDTA i.v. (25 one-day cycles)	Reduced (not specified)	[7]

56/M	Type 2 D	MOM^*∗*^	14–20 months	Prosthesis wear and hip dislocation	Hearing loss Sensorimotor disorders Walking difficulties Pericardial effusion Cardiomegaly Subclinical hypothyroidism	Yes	Co 506 mcg/L Cr 14.3 mcg/L	DMPS oral (14 mg/kg/day for 6 days, 4 mg/kg for 5 days, and 4 mg/kg for 4 days)	Reduced (not specified)	[8]

52/M	—	MOP^*∗*^	Not specified	Periarticular painful fluctuant mass with black fluid at aspiration	Dilated cardiomyopathy Pericardial effusion Liver failure Hypothyroidism EXITUS due to severe MOF	No	Co 1085 mcg/L Cr not reported	Dimercaprol (1 three-day cycle)	Co decrease (by 33%)	[9]

55/M	—	MOP^*∗*^	24 months	Myositis ossificans like picture	Visual/hearing loss Cardiomyopathy Hypothyroidism	Yes	Co 885 mcg/L Cr 48.8 mcg/L	DMPS (Not specified)	Not specified	[10]

75/M	—	MOM^*∗*^	60 months	Prosthesis wear and local metallosis	Asthenia Dilated cardiomyopathy Pericardial effusion	Yes	Co 46.5 mcg/L Cr 76.1 mcg/L	NAC oral + i.v. (i.v.: 150 mg/kg bolus + 300 mg/kg/d for 10 days) (Oral: 2 seven-day cycles at 100 mg/kg/d for each cycle)	Co/Cr decrease (by 51% and 40%)	[11]

67/M	Cochlear implant Hypert.	MOM	30 months	Little fluid collection near the acetabular cup	No	No	Co 22.5 mcg/L Cr 7.4 mcg/L	NAC oral (Oral 300 mg/kg/day for 10 days)	Co/Cr decrease (by 86% and 87%)	Case 1

81/F	COPD, SLE Mitral/Aort. reg. Renal imp. Euthyr. goiter Hypert., cataracts	MOM	65 months	Fluid collection near hip prosthesis	No	No	Co 21.1 mcg/L Cr 11.8	NAC oral (Oral 300 mg/kg/day for 9 days)	Co/Cr decrease (by 45% and 24%)	Case 2

M/F: male/female; Type 2 D: type 2 diabetes; Hypert.: hypertension; COPD: chronic obstructive pulmonary disease; SLE: systemic lupus erythematosus; Mitral/Aort. reg.: moderate mitral and aortic valve regurgitation; Renal imp.: mild renal impairment; Euthyr. goiter: Euthyroid multinodular goiter; Co: cobalt; Cr: chromium; MOP: metal on polyethylene; MOM: metal-on-metal; EDTA: edetate calcium disodium; DMPS: sodium 2,3-dimercaptopropane sulfonate; NAC: N-acetyl-cysteine; ^*∗*^the prosthesis has been implanted after the rupture of a previous ceramic hip implant.
